# Deep Learning-Enhanced Motor Training: A Hybrid VR and Exoskeleton System for Cognitive–Motor Rehabilitation

**DOI:** 10.3390/bioengineering12040331

**Published:** 2025-03-22

**Authors:** Kathya P. Acuña Luna, Edgar Rafael Hernandez-Rios, Victor Valencia, Carlos Trenado, Christian Peñaloza

**Affiliations:** 1 Mirai Innovation Research Institute, Osaka 559-0034, Japan; 2 Institute for the Future of Education Europe (IFE), 48014 Bilbao, Spain; 3 Institute of Clinical Neuroscience and Medical Psychology, Medical Faculty, Heinrich Heine University, 40225 Düsseldorf, Germany

**Keywords:** EEG, motor imagery, exoskeleton, machine learning, virtual reality, wavelet package decomposition, WPT

## Abstract

This research explored the integration of the real-time machine learning classification of motor imagery data with a brain–machine interface, leveraging prefabricated exoskeletons and an EEG headset integrated with virtual reality (VR). By combining these technologies, the study aimed to develop practical and scalable therapeutic applications for rehabilitation and daily motor training. The project showcased an optimized system designed to assess and train cognitive–motor functions in elderly individuals. Key innovations included a motor imagery EEG acquisition protocol for data classification and a machine learning framework leveraging deep learning with a wavelet packet transform for feature extraction. Comparative analyses were conducted with traditional models such as Support Vector Machines (SVMs), Convolutional Neural Networks (CNNs), and Long Short-Term Memory (LSTM) networks. The performance was further enhanced through a random hyperparameter search, optimizing feature extraction and learning parameters to achieve high classification accuracy (89.23%). A novel VR fishing game was developed to dynamically respond to EEG outputs, enabling the performance of interactive motor imagery tasks in coordination with upper limb exoskeleton arms. While clinical testing is ongoing, the system demonstrates potential for increasing ERD/ERS polarization rates in alpha and beta waves among elderly users after several weeks of training. This integrated approach offers a tangible step forward in creating effective, user-friendly solutions for motor function rehabilitation.

## 1. Introduction

In recent years, deep learning algorithms, virtual reality (VR), motor imagery brain–computer interfaces (BCIs), and exoskeleton robots have shown significant advancements, leading to increased reliability and rapid development. Combining these technologies has great potential, but enhancing their effectiveness and practicality for specialized applications is still in the early stages, especially in the biomedical field. Research studies and systematic reviews emphasize the importance and plausibility of such an integration while stressing its potential application in human enhancement and rehabilitation. This research proposes a multimodal system addressing the need for cognitive–motor rehabilitation in the elderly, considering current implementation challenges.

### 1.1. Multimodal Rehabilitation

#### 1.1.1. Virtual Reality (VR)

Previous studies targeting upper limb injury and disease recovery based on VR (multisensory high-end and game-based systems) reported non-inferiority to traditional rehabilitation techniques, while highlighting user motivation and satisfaction [1]. With regard to cognition, VR has been applied in attention rehabilitation for individuals suffering from stroke [2] or traumatic brain injury [3]. For cognitive–motor rehabilitation, previous efforts included training activities based on gaming, i.e., VR-based physical and cognitive training programs, that led to significant improvements in dual-task gait performance, which were attributed in turn to an enhancement in executive functions [4]. In the case of aging populations, it has been shown that VR can support the improvement or maintenance of cognitive functions by enhancing motivation for rehabilitation and cognitive abilities [5]. A positive effect of VR has also been reported on the executive functions of patients with mild cognitive impairment [6], stress management [7] and cognitive load [8]. Importantly, studies have shown that VR favors motor imagery responses during motor training [9,10].

#### 1.1.2. Exoskeleton Robots

It has been noted that through the use of an exoskeleton, exercise intensity progression correlates with changes in memory and gait endurance in Parkinson’s patients, which is in line with the hypotheses that high-intensity exercise improves physical and cognitive function via angiogenic and neurotrophic mechanisms [11]. In stroke patients, therapies with a free-standing exoskeleton have been shown to facilitate improvement in the level of independence and grip strength [12]. In particular, upper limb exoskeletons dealing with cognitive (through electromyography signals) and physical user interaction (through load cell sensors) led to the activation of neuroplasticity in patients suffering from neurological injury [13]. Meanwhile, other reports considered anatomical aspects, the joint range of motion, anthropometric parameters, disability assessment techniques, and robot-assisted training methods as well as cost efficiency [14,15]. Importantly, the role of exoskeletons has been emphasized in relation to motor imagery [16].

#### 1.1.3. Exoskeletons and VR

The interplay between exoskeletons and VR is particularly relevant in the field of cognitive and motor rehabilitation in combination with motor imagery [17,18,19]. It comprises aspects such as environment simulation (positioning/location of the exoskeleton, VR system), perception (multisensory), and feedback (user and device feedback) [20]. In particular, previous studies directed at motor training to control wearable lower limb exoskeletons for people with sensorimotor disorders emphasized that in order to maximize learning, users should train on sub-tasks sequentially using the most suitable combination of perception and visual feedback for each sub-task [21]. Recently, an innovative mirror therapy system was also introduced that pairs a VR system and a soft hand exoskeleton, physically inducing the mirrored motion in the real hand. The system was well tolerated, without adverse events, and provided an appropriate sense of embodiment [22]. Another study targeting rehabilitation in brain-injured patients integrated a lower limb exoskeleton, a VR game system, and an EEG motor imagery brain–computer interface (MI-BCI) based on EEGNet. The authors reported a model accuracy of 85.2% for MI detection and 78.3% for classification (offline session) and an accuracy of 67.2% (pseudo-online session), although participants were only able to complete the game with difficulties in game control during the online session [23]. A pilot study conducted on a single subject considered the use of an interface between a hand exoskeleton system (HES) and variable admittance control to achieve VR-based rehabilitation tasks. The exoskeleton assisted hand movements according to force feedback and a reference value calculated inside the VR environment. Whenever the subject grasped a virtual object, the HES provided the user with a force feedback sensation [24]. A clinically validated study addressing performance in a reaching task carried out by stroke patients used an upper limb force feedback exoskeleton for robotic-assisted rehabilitation in virtual reality (VR). The evaluation showed a significant reduction in the performance error in the task [25]. Other authors integrated virtual reality (VR) goggles and a motor imagery (MI) brain–computer interface (BCI) algorithm with a lower limb rehabilitation exoskeleton robot (LLRER) system. The experimental results showed that the VR goggles had a positive effect on the classification accuracy of the MI-BCI. The best results were obtained with subjects in a seated position wearing the VR goggles. The classification model for seated VR use had an accuracy of 75.35% in the open-loop test of the LLRER, and the accuracy of correctly triggering the rehabilitation action in closed-loop gait rehabilitation of the LLRER was 74% [26].

### 1.2. Proposed Solution

Based on the above-mentioned, the integration of deep learning, VR, and an upper limb exoskeleton robot has the potential to address increasingly complex rehabilitation and assistive needs more effectively than isolated solutions. BCI-VR integration enhances key human–machine interaction processes, such as recognition, perception, and feedback [9]. Moreover, VR’s ability to provide rich immersion and illusions through egocentrically simulated virtual scenarios has been shown to enhance rhythmic patterns and improve spatial feature discrimination compared to monitor displays. This, in turn, improves motor imagery training [27] and cognitive–motor training, for instance, in the elderly [28,29]. Such an improvement in brain signal classification leads to the improved control of exoskeleton robots assisting subjects in completing various rehabilitation exercises that favor motor abilities. Consequently, this research proposes a holistic integration of all these technologies, aiming to create a more comprehensive and effective rehabilitation tool. This integrated approach is particularly pertinent for addressing the multifaceted cognitive and motor training needs of the elderly population, highlighting the necessity for more rigorous, high-quality research in this domain.

As the world’s population is rapidly aging, there has been a significant increase in the prevalence of age-related cognitive decline. This demographic shift underscores a substantial social and economic imperative to develop systems that can facilitate independence and autonomy for the elderly [29]. It is estimated that by 2050, approximately 21% of the global population will be aged 60 or over [30], heightening the demand for effective interventions to maintain independence and quality of life among older adults. Several studies have revealed significant neuroplastic effects in four main cognitive functions in the elderly, i.e., visuospatial skills, language, memory, and their intellectual capacity, resulting from as few as five sessions of motor imagery training [31]. Other studies, such as that by Buranova et al. [32], have highlighted the potential benefits of motor imagery as a therapeutic tool in healthy aging, particularly for those with restricted mobility. These studies also suggest a link between increased motor-related beta desynchronization (MRBD) and motor decline, with older adults exhibiting stronger MRBD during motor execution (ME) and motor imagery (MI) due to increased GABAergic inhibition.

We aim to show the plausibility of an integrated, noise-resistant VR–exoskeleton–EEG system for the real-time classification of motor imagery data tested on a group of ten healthy subjects. The proposed system serves as a proof of concept for future, more extensive studies targeting the improvement of motor abilities in elderly groups. In particular, our goals included (a) the integration of all system components in a robust configuration to minimize errors and maximize accuracy, (b) the optimization of a machine learning algorithm for motor imagery training using real-time EEG data, and (c) the development of a VR game that is coupled with two arm exoskeletons for motor training, specifically designed for elderly patients.

Two neurophysiological measures, i.e., event-related desynchronization (ERD) and event-related synchronization (ERS) in the mu and beta brainwave frequencies, are proposed as the system outputs. Mu and beta frequencies are relevant for assessing brain changes in relation to motor imagery and serve as valuable indicators of neuroplasticity resulting from motor training [33]. Monitoring ERD and ERS allows the system to evaluate the rehabilitation effectiveness. This provides insights into neural adaptations and motor function improvements in elderly patients. The mu rhythm, corresponding to a frequency of 8–12 Hz, is typically observed over the sensorimotor cortex and decreases during both actual movement and motor imagery, reflecting the engagement of the motor cortex. The beta rhythm, corresponding to a frequency of 13–30 Hz, shows similar desynchronization during motor imagery and is often linked with active, focused states and motor control. ERD refers to a decrease in the amplitude of EEG rhythms in response to a specific event or stimulus, while ERS refers to an increase in the EEG rhythm amplitude. Importantly, ERD and ERS reflect changes in the activity of local interactions between main neurons and interneurons that control the frequency components of the ongoing EEG [33]. ERS often follows ERD and can be observed in similar frequency bands, particularly in the beta band during motor tasks.

Unlike conventional rehabilitation devices that separately employ virtual reality (VR), brain–computer interfaces (BCIs), or exoskeletons, our system introduces a fully integrated, real-time framework that synergistically combines these technologies with deep learning-based classification. Prior studies have explored VR-driven cognitive–motor rehabilitation and exoskeleton-assisted therapy, but they have often lacked robust adaptability to user-specific neurophysiological patterns or relied on pre-programmed movement sequences. In contrast, our approach leverages real-time EEG-based motor imagery detection with wavelet packet transform (WPT) feature extraction, achieving a high classification accuracy of 89.23%. This not only enhances system responsiveness but also enables personalized rehabilitation by dynamically adjusting VR–exoskeleton interactions based on individual neural activity. By integrating these elements into a gamified training environment, our system advances rehabilitation research, offering a more immersive, adaptive, and data-driven solution for improving motor function tailored to elderly users.

## 2. Materials and Methods

### 2.1. Hardware and Software

The realization of the proposed integrated framework was made possible by using a workstation with a CPU (Intel Core i7, Intel, Santa Clara, CA, USA), GPU (NVIDIA GTX 2050 Ti, NVIDIA, Santa Clara, CA, USA), and 16 GB DDR4 RAM (Intel, Santa Clara, CA, USA) and storage (1 TB SSB), which ensured efficient real-time processing for EEG signal acquisition, VR rendering, and machine learning computations.

For the EEG, AURA software (https://www.mirai-innovation-lab.com/aura-through-time/, accessed on 17 March 2025) was used for real-time EEG data processing and the WAVEX system (https://www.mirai-innovation-lab.com/wavex/, accessed on 17 March 2025) for EEG acquisition. WAVEX is an EEG system with 8 channels that samples at a 250 Hz frequency. The LabStreamingLayer (LSL) (https://labstreaminglayer.org/#/, accessed on 17 March 2025) was useful for streaming EEG data in real time. Python 3.10 with TensorFlow (https://www.tensorflow.org/, accessed on 17 March 2025), PyTorch (https://pytorch.org/, accessed on 17 March 2025), and Scikit-learn (https://scikit-learn.org/stable/, accessed on 17 March 2025) was used for the machine learning algorithms.

Unity 2022.3 was used for the development of the VR environment (environment simulation and real-time interaction). C# was used for scripting and event-driven interactions. For the real-time synchronization of EEG, VR, and exoskeleton control, the LabStreamingLayer (LSL) was employed, and finally a UART via an Arduino MEGA was used to communicate with the exoskeleton.

In addition to the Arduino MEGA, the exoskeleton was controlled with DC motors and encoders. The UART (serial communication) sent movement commands from the VR system to the exoskeleton.

The described setup components ensured that EEG-based movement intentions were effectively translated into real-time exoskeleton actions.

### 2.2. Project Stages

The development of this project can be divided into the following stages: (1) the design of a data acquisition protocol and subsequent data collection using EEG, (2) feature extraction and deep learning algorithm design, (3) the development of the VR game, (4) system integration including the exoskeletons, the VR game, and real-time classification using the DL algorithm, and (5) data analysis.

#### 2.2.1. Data Acquisition Protocol

**Figure 1:**
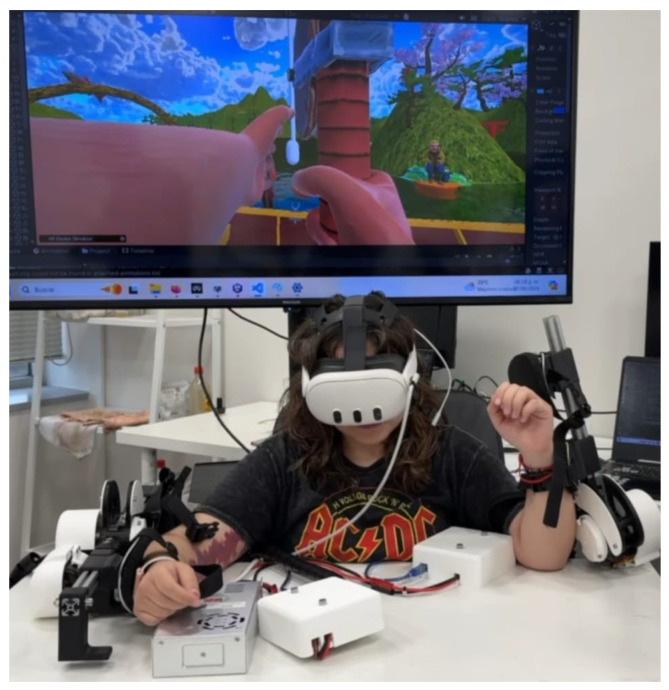
Full VR–exoskeleton–EEG system on a user.

The proposed data acquisition protocol consisted of two sessions with a two-week difference between sessions. This time difference aimed to eliminate any event-specific bias in the data, such as specific events, or physical and mental fatigue that may have interfered with the data quality. Training data were collected from ten healthy participants aged between 20 and 50 using a Wavex 3 headset, which incorporated an 8-electrode EEG, and an Oculus Quest 3 [34]. The socio-demographic data of the participants can be seen in Table 1. A custom Wavex headset was crafted to ensure electrode placement consistency, focusing on the Supplementary Motor Cortex area. This research acquired preliminary data using a standard Wavex headset; the whole system can be seen in Figure 1.

The first session aimed to acquire training data and was performed entirely offline, while the second session was used for validation purposes. Satisfaction surveys were implemented at the end of each session to measure satisfaction or frustration with the protocol. During the second session, an additional survey was added to evaluate the perceived accuracy of the VR–exoskeleton response to movement intentions. The full results and content of this survey can be found in Appendix A.

The data acquisition consisted of two active modules, the first dealing with real movement (RM) and the second with motor imaginary (MI) movement. Rest modules were included in between repetitions. There were ten repetitions per module to acquire sufficient data. A break period was included to avoid mental or physical fatigue. The instruction for the rest module was to focus on tongue movement, which has been reported as a technique to suppress the amplitude of the arm area mu rhythm by consciously directing attention to different body parts or limbs [33]. A simple Python interface with timers and visual instructions was constructed to facilitate interaction with the participant and save the acquired EEG data in a csv format.

The data acquisition protocol had the following final structure:

- Preparation—15 min.
- Electrode placement—10 min.
- Training explanation—5 min. Included a brief overview of the modules and instructions for the trial subject.
- Rest module—10 s.
- RM module (using visual cue interface)—7 min (repeated 10 times).
  – Left arm.
  – Flex—5 s.
  – Extend—5 s.
  – Right arm.
  – Flex—5 s.
  – Extend—5 s.
- Rest break (no data were collected at this stage).
- MI module (using visual cue interface)—7 min (repeated 10 times).
  – Left arm.
  – Flex—5 s.
  – Extend—5 s.
  – Right arm.
  – Flex—5 s.
  – Extend—5 s.
  – Rest—10 s.

The total estimated time for each session was 30 min.

The raw EEG data were obtained through the Aura software interface [35], which transmitted the information using the lab-streaming layer protocol (LSL) [36]. This protocol was synchronized with the data acquisition interface, operating at a sampling rate of 100 Hz. The data were then labeled with one of five possible module categories: (1) right arm extension, (2) right arm flexion, (3) left arm extension, (4) left arm flexion, or (5) rest. The EEG data recorded information from each of the eight electrodes across five brainwave frequencies, delta, theta, alpha, beta, and gamma, resulting in a total of 40 variables.

To minimize excessive sources of noise, participants were evaluated in a room with minimal distractions and instructed to avoid unnecessary body movements (head and facial movements) as much as possible. Each data acquisition cycle lasted only 5 to 10 s and was manually activated by the researcher, allowing for pauses in data collection if the participant indicated the need to shift positions. One study limitation was the lack of an electrooculogram (EoG) and other sensing devices for noise filtering. However, the use of sensors can be easily incorporated into the software pipeline in the future. Importantly, the proposed system aims to be clinically feasible in less-than-ideal noise conditions such as the ones presented during this data collection, which is why noise minimization was mainly addressed during data preprocessing, particularly by using wavelet packet decomposition (WPD).

WPD breaks down the EEG signal into components in different frequency bands, enabling the precise isolation of relevant brainwave frequencies and filtering out unrelated noise. In addition, it captures both the transient and steady-state components of EEG signals, which has been shown to improve the robustness of motor imagery classification in noisy environments and improve the signal-to-noise ratio. It is important to note that despite its benefits, the WPT may not fully address noise from electrode movement artifacts, high-amplitude external electromagnetic interference, and physiological noise such as heartbeats and respiration.

#### 2.2.2. Deep Learning Training

The data acquired from the ten subjects were concatenated and normalized to be used as an input to the deep learning algorithm. Based on a thorough analysis of current research in EEG data feature extraction, the wavelet packet transform (WPT) was selected as the optimal technique [37,38]. WPD was implemented using multilevel decomposition using wavedec (pywt.wavedec) from the PyWavelets library [39]. This function offers a multilevel 1D Discrete Wavelet transformation of data that can break down the input data into various frequency components at multiple levels of detail, which is valuable for capturing different aspects of the signal.

The key hyperparameters for this method are the wavelet and level. The flexibility in wavelet selection allows for specifying different types of wavelets (e.g., ‘db1’, ‘haar’, ‘sym2’, etc.). The choice of wavelet can impact the features extracted and, consequently, the performance of the model.

On the other hand, the level specifies the number of levels of decomposition of the signal analyzed. For a given level, *n*, the output of pywt.wavedec is a list of arrays:

- cA_n: Approximation coefficients at level *n*.
- cD_n: Detail coefficients at level *n*.
- cD_(n-1): Detail coefficients at level n−1.
- …
- cD_1: Detail coefficients at level 1.

The coefficients can be interpreted as follows [40]:

- Approximation Coefficients (cA_n): These represent the low-frequency, coarse structure of the signal at the highest level of decomposition and may capture the overall trends or baseline activity in EEG signals.
- Detail Coefficients (cD_n, cD_(n-1), …, cD_1): These represent the high-frequency details of the signal at various levels of decomposition. Each set of detail coefficients captures progressively finer details. They can capture transient events, oscillations, and other fine-grained features that might be relevant for tasks like the classification of cognitive states or detection of abnormalities.

The number of levels and the wavelet are selected based on the characteristics of the signal and the specific analysis goals. Common practice might involve 3 to 5 levels of decomposition to capture the relevant frequency bands (e.g., delta, theta, alpha, beta, and gamma).

A random hyperparameter search to determine the WPT considering these two key hyperparameters was implemented at the beginning of every training iteration to enhance this methodology’s flexibility and ensure the reproducibility of the results. This approach aimed to optimize the code for various motor imagery tasks, which involved high variability in real-time EEG data from a diverse cohort of subjects.

The algorithm was subsequently saved as a pre-trained model for real-time data classification. This real-time machine learning code classified the EEG input into five distinct states, subsequently triggering appropriate responses in both the exoskeleton and the VR game. These responses provided the user with synchronized visual and proprioceptive feedback.

The Convolutional Neural Network (CNN) was developed using the Keras library within TensorFlow [41]. Prior to the WPT hyperparameter search, a separate random hyperparameter search was conducted to determine the optimal Keras model parameters, which were statically defined before proceeding with the WPT search. The hyperparameters considered in this grid search included the following:


param_grid = {

‘batch_size’: [10, 20, 30],

‘epochs’: [5, 10, 20],

‘optimizer’: [‘adam’, ‘rmsprop’],

‘init_mode’: [‘uniform’, ‘lecun_uniform’,

          ‘normal’],

‘activation’: [‘relu’, ‘tanh’],

‘neurons’: [32, 64, 128]

}


The WPT hyperparameter search was designed to maximize accuracy, after which the optimal hyperparameters were selected to train and save the complete deep learning algorithm. There were three wavelet types considered by the hyperparameter search based on their suitability for EEG signals, whose characteristics vary between acquisitions. Daubechies (db4) are considered to have a good balance between time and frequency localization; however, since they are not symmetric, they can introduce phase distortion during reconstruction and are more sensitive to noise than other methods. Coiflets (coif1) have near-symmetric waveforms and reduce phase distortion but are more complex, making model training harder and computationally expensive, especially when working with little data. Symlets (sym5) are a symmetrical variation of Daubechies very suitable for minimizing reconstruction errors, balancing the characteristics of Daubechies and Coiflets, although with diminished time localization [40].

The classification model’s accuracy with different wavelets and decomposition levels, as well as other hyperparameters, was assessed through cross-validation using 5 K-folds, with the accuracy averaged across folds to determine the best combination of the wavelet types and levels. Subsequently, a confusion matrix was generated to compare the performance of the deep learning algorithm with other conventional methods. The research compared the results with those obtained from a Long Short-Term Memory (LSTM) network, a Support Vector Machine (SVM), and a Random Forest classifier.

#### 2.2.3. VR Game Development

The development of the virtual reality (VR) game for motor training was guided by several crucial aspects to enhance both user engagement and the effectiveness of the motor imagery exercises. The following primary focus areas were prioritized according to the literature-based evidence and participant feedback from non-VR EEG data acquisition:

- Visual Feedback for Motor Imagery: To support the motor imagery process, the game includes visual cues that correspond to the imagined or executed movements. These cues were designed to be intuitive and closely align with the user’s intended actions, thereby enhancing the mental and physical connection.
- Color and Sound Cues: The integration of color and sound cues plays a significant role in improving the clarity of instructions and actions within the game. High-contrast colors are used to highlight important elements, while sound cues provide immediate auditory feedback, contributing to an immersive and clear user experience, which is especially relevant to elderly audiences who might be unfamiliar with the technology.
- Engaging Storytelling: The game incorporates a narrative element that is both engaging and appropriate for the elderly target demographic. This storytelling aspect not only makes the game more enjoyable but also helps to maintain user motivation and focus during training sessions.
- Balance Between Simplicity and Realism: A critical balance is maintained between simplicity and realism in the game’s design. While the game is easy to navigate and understand, it also provides a realistic simulation of motor tasks to ensure that the training translates effectively to real-world activities.

**Figure 2:**
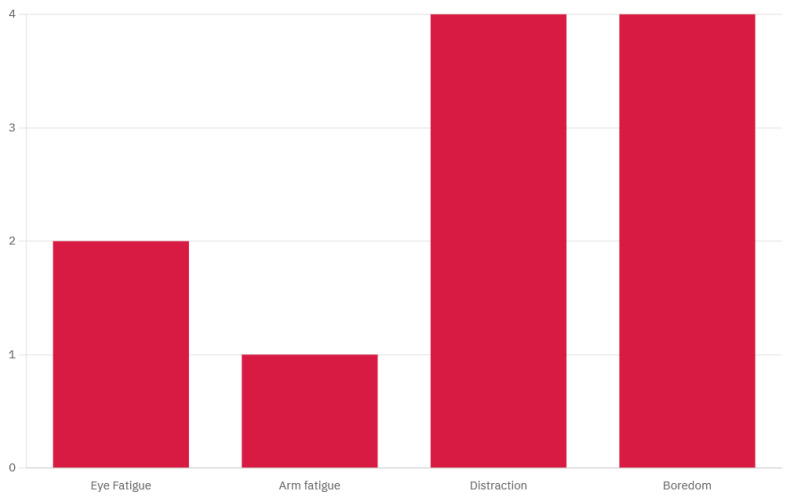
User complaints during EEG data acquisition without VR.

These aspects were grounded in the literature that supports their effectiveness in VR-based motor training for elderly users [42] and were further validated by feedback received during the first data acquisition session survey. The full survey data are available in Appendix A, and the results are presented in Figure 2.

The game’s design prioritizes a user-friendly interface that is intuitive and easy to navigate, minimizing the cognitive load and ensuring accessibility for users with minimal technological experience. The aesthetic is calming and minimalistic, with visual and auditory elements carefully crafted to avoid overstimulation and foster a soothing environment conducive to focus and relaxation. A high color contrast is employed to enhance readability, particularly for users with age-related visual impairments, while the slow-paced interactions accommodate the reaction times of elderly users, preventing any sense of being rushed. The environment is brightly lit, creating a sense of openness and positivity, further contributing to user comfort. Additionally, the game incorporates familiar elements with positive connotations, such as nature scenes, to evoke pleasant emotions and enhance the overall user experience.

#### 2.2.4. VR Mechanics

**Figure 3:**
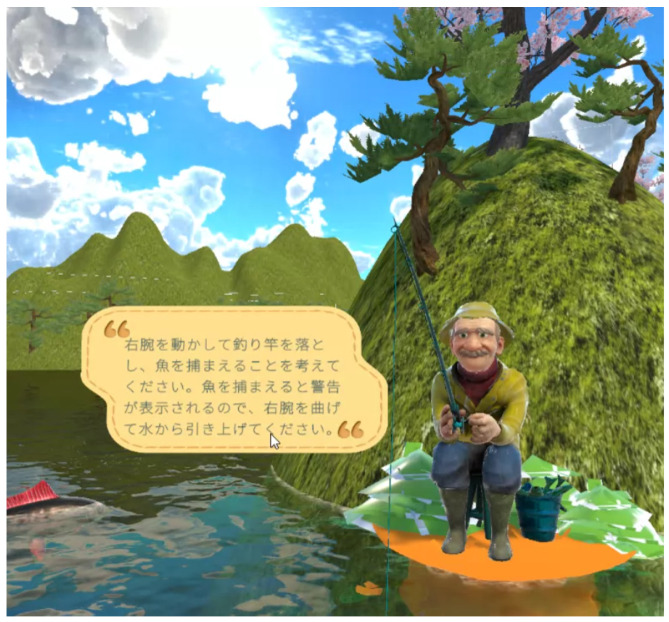
VR game: Introduction scene.

The core mechanics of the VR game involve a simple yet engaging fishing activity. The user is seated in a virtual boat, simulating a calm, stationary environment that minimizes physical strain (Figure 3). The mechanics were designed as follows:

- Right Arm Flexion/Extension: The user’s right arm controls the swing of a fishing rod. The flexion and extension movements are tracked and translated into the corresponding actions of casting and swinging the rod.
- Left Arm Flexion/Extension: The left arm is responsible for picking up the fish caught on the rod and dropping it into a basket. This action requires coordination and spatial awareness, as the game aligns the rod, basket, and fish in a manner consistent with real-world perceptions of object separation and depth.

**Figure 4:**
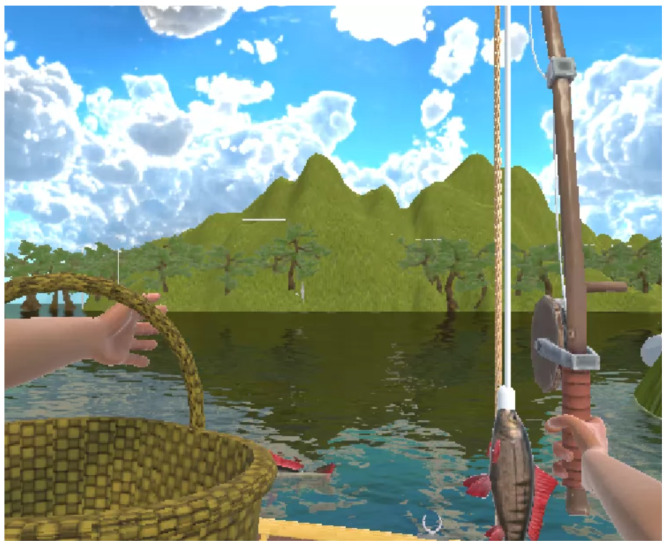
VR game: Initial state of the upper limb motor task.

The game operates according to the following rules:

- Initial State: The user begins seated in a boat with their right arm flexed, holding the rod vertically, and their left arm extended (Figure 4).
- Dropping the Rod: Extending the right arm causes the rod to drop into the water.
- Hooking the Fish: After the rod enters the water, a fish may bite after a random interval of between 5 and 10 s. This is indicated by a noticeable downward pull on the rod, which may be accompanied by an audio or visual cue.
- Reeling In: The user must flex their right arm to reel in the rod and catch the fish within 5 s of the hooking animation starting. If the user fails to reel in the rod within this time frame, the animation will stop, and another random interval must pass before a new fish bites.
- Unhooking the Fish: To unhook the fish, the user should flex their left arm, which will automatically cause the hand to grasp the fish. The right arm must remain stationary; if it extends before unhooking is complete, the fish will return to the water, requiring the user to wait for another hooking opportunity.
- Dropping into the Basket: Extending the left arm again will cause the fish to drop automatically into the basket. At this stage, the movement of the right arm is irrelevant; flexing or extending it will not affect the process.

#### 2.2.5. System Integration

The system integration involved the real-time classification of EEG data using the pre-trained machine learning algorithm to control the flexion and extension of two repurposed exoskeleton arms, as well as the arm controllers within the VR game environment. The materials used for this system included a Wavex EEG headset paired with an Oculus Rift 3 for immersive virtual reality interaction. Wavex is a customizable EEG headset developed by MIRAI Innovation and is coupled with their AURA software for signal recording and processing. The exoskeletons employed in this setup were Alice OSE (Open Source) Exoskeleton Models developed by Indi, which were adapted to function as arm exoskeletons. The Alice exoskeleton is a low-budget exoskeleton with one degree of freedom supported by the Arduino IDE and works with a 12 V power source [43].

The EEG signals were acquired by the algorithm through LSL transmission, facilitated by the AURA software, and subsequently processed by the pre-trained model. These signals were then classified to identify the intended actuator triggers. The classified output was simultaneously transmitted via the LSL to Unity [36], where it was used to control the VR environment, and through Arduino serial port communication for the precise actuation of the exoskeleton. This dual-path transmission ensured that both the VR interface and the exoskeleton movements were synchronized, providing a cohesive and responsive user experience.

**Figure 5:**
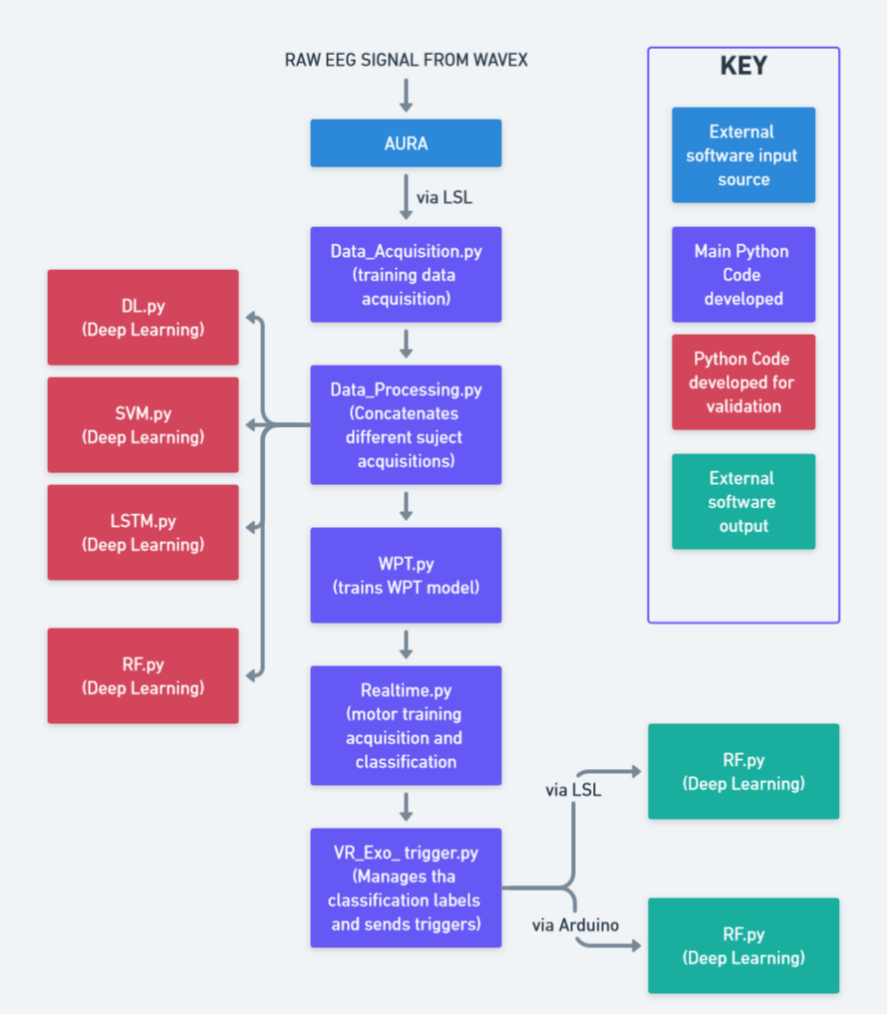
System integration diagram.

**Figure 6:**
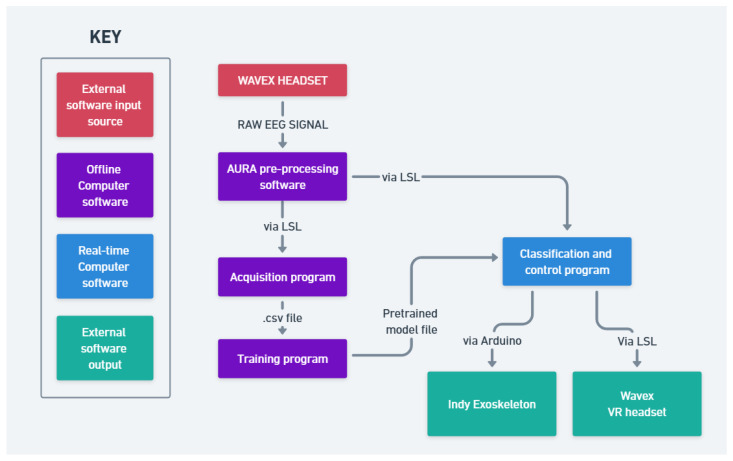
System hardware diagram.

The full schematic detailing the relationship between different modules of the project is shown in Figure 5 and Figure 6.

### 2.3. Data Analysis

Finally, a Python program was developed to systematically assess the ERD and ERS responses derived from the data acquired through the data acquisition protocol, both before and after motor training sessions. The ERD and ERS were calculated by analyzing the amplitude of EEG signals in each training cycle and for each task by considering the mu and beta frequency bands, differentiating by the hemisphere based on the electrode placement. For each data train, an average was calculated to serve as a baseline. Then, the maximum amplitude changes, both increases and decreases, between individual samples were detected as an indicator of the ERD/ERS polarization strength. An increase in the amplitude and a wider ERD/ERS ratio can indicate progress in motor imagery training, neuroplasticity, or recovery from motor impairments. In the context of elderly patients, it can also indicate improved motor skills and healthy aging.

The data analysis output focused on the measurement of ERD and ERS amplitude changes while discriminating between the data in the following categories: (a) the left arm and right arm, (b) extension and flexion, (c) left hemisphere and right hemisphere electrodes, (d) alpha and beta brainwaves, and (e) repetition cycle (1 to 10). These categories allowed for a flexible and granular analysis of the acquired data, facilitating the study of the brain in relation to human motor control. This approach will allow for adaptability in future research endeavors, accommodating different research questions from different areas of interest. This analysis is crucial for quantifying the neurophysiological changes induced by motor imagery training, particularly its effectiveness in mitigating motor decline. The code was designed to process the EEG data by extracting relevant features associated with mu and beta rhythms, which are known indicators of motor cortex activity. By comparing the ERD/ERS patterns pre- and post-intervention, the program provides a means to validate the effectiveness of the training protocol.

## 3. Results

The project successfully demonstrated the functionality and reliability of the proposed integrated system. The results obtained serve mainly as a flexible proof of concept, validating the system’s ability to synchronize exoskeleton movements with VR responsiveness in accordance with the intended motor imagery tasks. The coordination between EEG signal classification, immersion provided by the VR environment, and exoskeleton control was confirmed to be consistent and effective, with minimal noise detected in the acquired data. To further assess the system’s effectiveness, a final satisfaction survey was conducted with a trial subject, focusing on the perceived accuracy of the VR–exoskeleton response to movement intentions. The survey results indicated an adequate level of satisfaction, affirming the system’s responsiveness and alignment with the user’s intended actions.

**Figure 7:**
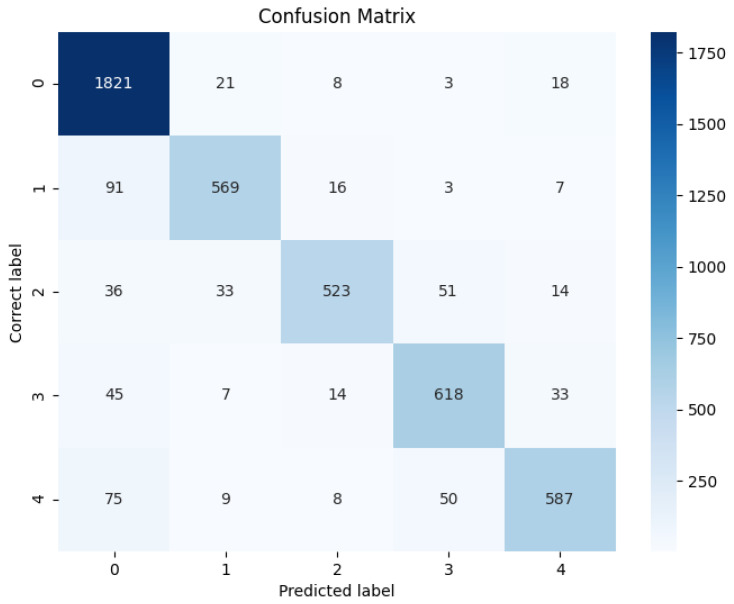
Confusion matrix for WPT model.

The proposed deep learning algorithm, along with the comparative machine learning models, achieved the following maximum accuracies on the training data (see also Figure 7):

- Random Forest: 60.83%.
- SVM: 57.86%.
- LSTM: 78.1%.
- Deep Neural Network: 71.08%.
- Wavelet Package Neural Network: 89.23%.

The following figures exemplify the preset automatic data analysis of the motor training protocol (RM module). It is important to clarify that the acquisition of data was performed during an initial session with healthy young individuals to assess the system’s feasibility and does not represent clinical outcomes.

**Figure 8:**
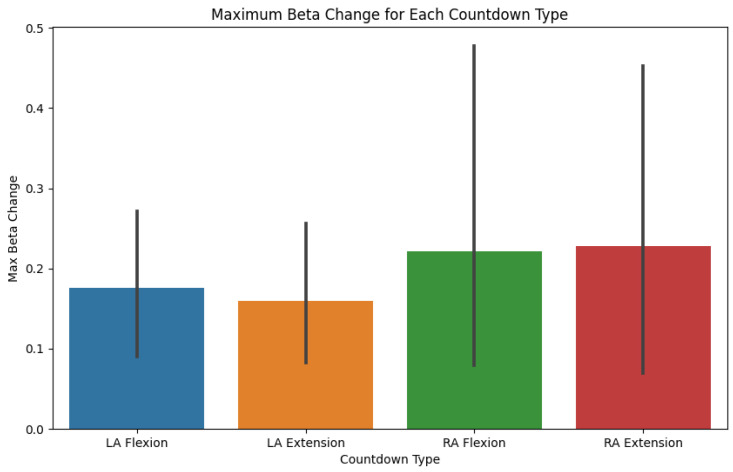
ERD/ERS ratio differential graph sample.

**Figure 9:**
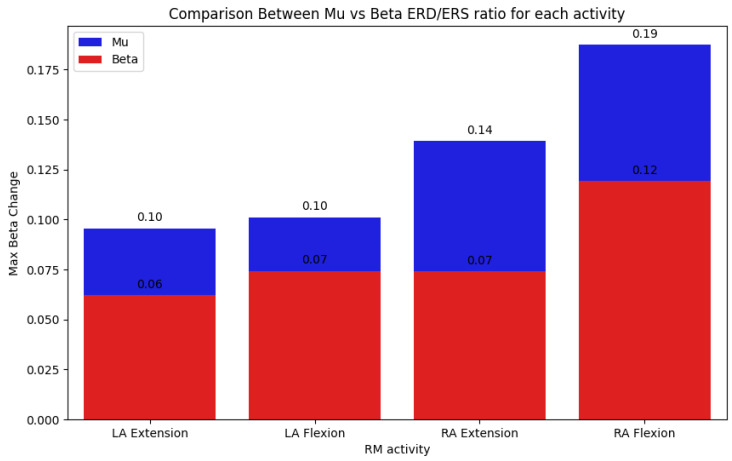
ERD/ERS graph sample.

Figure 8 shows the maximum beta change corresponding to each task. Figure 9 presents the comparison between the ERD/ERS ratios at the mu and beta frequency bands for each of the movements performed: left arm extension, left arm flexion, right arm extension, and right arm flexion.

## 4. Discussion

The present study was exploratory in the sense that no training protocol, sample size calculation, and hypothesis concerning the effect of training on the system-induced brain plasticity were formulated beforehand. As such, the results presented are qualitative in nature; nevertheless, our pilot study provides relevant insights into the system parameters and properties (optimal classification algorithm, immersion provided by the VR environment, the comfort of the EEG/VR head set and exoskeleton robot, the sense of embodiment, the variability of physiological measures across tasks and participants, the duration of experimental sessions, etc.) that support the design of future protocols for elderly populations.

The testing of the proposed integrated system was conducted on a cohort of ten healthy individuals. Participants reported that the system was well tolerated and caused no dizziness or physical discomfort and that the created environment enhanced motivation for and engagement in performing the task while being more ecologically valid than traditional experiments based on stereotypical tasks.

Our results demonstrated the feasibility of a machine learning algorithm to successfully classify motor imagery states, even when faced with noisy or limited data. Our classification algorithm (Wavelet Package Neural Network) showed a higher accuracy of 89.23% in comparison to previous studies also making use of motor imagery [23,26]. In particular, the system’s robustness and adaptability were showcased, which are crucial for real-world applications where data conditions may vary significantly. The flexibility of the algorithm also extends to its ability to accommodate changing databases and data formats, ensuring long-term usability and adaptability in diverse research and clinical settings. Additionally, the development of a VR game tailored to user needs, and its integration with both the exoskeleton and Wavex headset, underscores the system’s responsiveness and the potential for enhancing user engagement during motor training. The real-time interaction between the VR game and the exoskeleton highlights the seamless integration of the components, which is vital for effective motor imagery tasks.

In contrast to previous MI studies addressing lower limb motor training and gait in patients suffering from neurological disorders [23,26], our system is directed toward the cognitive and motor rehabilitation of elderly populations by using an upper limb exoskeleton that facilitates hand movement during an ecologically valid task, namely a fishing task. Importantly, our system proposes the use of measures (ERS and ERD) to assess brain plasticity changes induced by training. As evidenced by the obtained results, such measures showed differences in their amplitude across different motor tasks and subjects, highlighting inter-subject variability training effects.

A key limitation of the current system is its susceptibility to EEG noise, particularly from muscle activity, eye movements, and environmental interference. While the wavelet packet transform (WPT) helps mitigate some artifacts, additional filtering methods, such as independent component analysis (ICA) or adaptive noise cancellation, should be explored in future iterations. The absence of electrooculogram (EoG) sensors in this study further limited precise eye movement artifact removal. Future implementations should incorporate multimodal sensing strategies to enhance the signal quality and ensure robust classification performance across diverse conditions.

Moreover, integrating EEG data with VR and exoskeleton control presents hardware challenges, including synchronization delays and computational load balancing. The reliance on the LabStreamingLayer (LSL) for real-time data transmission, while effective, introduces potential latency issues that may affect response accuracy. Ensuring seamless real-time communication between these components requires the optimization of transmission protocols and embedded processing solutions, which will be critical for improving the system stability in clinical applications. Investigating alternative wireless communication methods and refining motor command execution in the exoskeleton could further reduce delays and enhance the overall user experience. Addressing these challenges will be vital for transitioning from feasibility testing to real-world clinical deployment.

Future work will be directed toward testing a cohort of healthy elderly subjects and evaluating the system’s efficacy in improving cognitive and motor abilities. It is expected that training the classification model with a larger dataset would improve its accuracy and reliability, particularly in noisy environments and uncertain conditions. Enhancing VR gamification elements could further increase user engagement, which is especially important for sustained therapeutic use. In addition, coupling data acquisition with VR gaming could expand the applicability of the proposed system from training to a more comprehensive assessment tool, potentially leading to more accurate evaluations of motor responses in settings that closely resemble real-life environments. The validation of the system with more ergonomic exoskeleton designs is another critical step, ensuring that the technology is not only effective but also comfortable and accessible for elderly users. Lastly, expanding the participant pool for data quality assessment and user satisfaction surveys will provide a more comprehensive understanding of the system’s effectiveness and areas for improvement.

To further validate the efficacy of the system, proposed future studies would incorporate preliminary clinical trials with elderly participants undergoing extended rehabilitation sessions. A controlled experimental design, comparing system-assisted training with traditional rehabilitation methods, could assess improvements in motor function, neuroplasticity markers, and user engagement. Longitudinal studies measuring event-related desynchronization/synchronization (ERD/ERS) changes over weeks or months would provide deeper insights into the system’s long-term impact. Additionally, testing the system with individuals recovering from stroke or neurodegenerative conditions could determine the system’s adaptability to clinical populations.

## 5. Conclusions

The proposed system offers a significant contribution to the existing body of knowledge on the integration of virtual reality (VR), exoskeletons, and EEG technology for motor training, with a particular focus on elderly rehabilitation. The results demonstrate that the system achieves robust and accurate classification using accessible components, offering a tool for the research and development of motor training protocols tailored to elderly patients. The system’s ability to minimize noise and ensure data replicability underscores its potential utility in further studies aimed at advancing motor imagery interventions. Despite the successful development and implementation of this integrated tool, the study was constrained by the limited size of its training data sample. Additionally, the absence of direct testing with the target demographic leaves some uncertainty regarding the system’s optimization and its impact on ERD/ERS polarization. As such, while the findings are promising, they should be regarded as preliminary, necessitating further exploration and validation. Furthermore, improving signal processing techniques, such as advanced noise filtering and adaptive artifact rejection, will be crucial to enhancing the classification accuracy across diverse environments. Addressing hardware integration barriers, particularly in optimizing real-time synchronization between EEG, VR, and exoskeleton control, will be another critical area of development. Refining exoskeleton movement precision and response times will further enhance the system’s usability for rehabilitation. These advancements will be necessary to bridge the gap between proof-of-concept validation and a fully deployable clinical solution. This research, nevertheless, lays a strong foundation for future advancements in this interdisciplinary field, addressing existing gaps and providing a promising framework for the development of more practical therapeutic solutions. Future efforts should focus on larger-scale user trials, particularly with elderly and neurologically impaired individuals, to assess the real-world applicability and therapeutic benefits.

## Figures and Tables

**Table 1:** Demographic information of participants.

Subject Number	Age	Gender
Sub-1	24	Female
Sub-2	27	Female
Sub-3	25	Male
Sub-4	24	Male
Sub-5	20	Male
Sub-6	29	Male
Sub-7	28	Male
Sub-8	32	Male
Sub-9	49	Female
Sub-10	25	Male

## Data Availability

The original data presented in the study are openly available in the following GitHub repository: https://github.com/Pauliebre/Toph_ML, accessed on 17 March 2025.

## Abbreviations

The following abbreviations are used in this manuscript:

BCI	Brain–computer interface
VR	Virtual reality
MRBD	Motor-related beta desynchronization
ERD	Event-related resynchronization
ERS	Event-related synchronization
CNN	Convolutional Neural Network
WPT	Wavelet package transform

## Appendix A. EEG Data Collection Satisfaction Surveys

Survey Question: Which of the following options were you significantly experiencing and were a problem during your EEG data recording session? (Select multiple choices if relevant).

**Table A1:** Participant answers for simple visual interface EEG data collection.

Participant	Eye Fatigue	Arms Fatigued	Distraction	Boredom
Sub-1	Yes	Yes	Yes	Yes
Sub-2	Only at the end	No	No	No
Sub-3	No	No	Yes	Yes
Sub-4	No	No	Yes	Yes
Sub-5	No	No	Yes	Yes
Sub-6	No	No	Yes	No
Sub-7	No	No	No	No
Sub-8	Only at the beginning	No	No	No
Sub-9	No	No	No	No
Sub-10	No	Yes	Yes	No
